# Draft genome sequences of eight bacterial isolates from *Pisum sativum* leaf surfaces

**DOI:** 10.1128/mra.00813-23

**Published:** 2023-12-20

**Authors:** Melanie R. Smee, Kathryn L. Herr, Sakinah Abdul-Khaliq, Chris E. A. Cadet, Tory A. Hendry

**Affiliations:** 1 Department of Microbiology, Cornell University, Ithaca, New York, USA; Rochester Institute of Technology, Rochester, New York, USA

**Keywords:** phyllosphere, epiphytic, legumes, synthetic community, host-microbe, plant-microbe interactions

## Abstract

Bacterial communities in the phyllosphere, the above-ground parts of plants, are diverse yet understudied. These bacteria are important for plant health and also for inter-kingdom interactions with beneficial and pest insect species. Here, we present draft genomes of eight culturable bacterial isolates from leaf surfaces in the *Pisum sativum* phyllosphere.

## ANNOUNCEMENT

The phyllosphere, the above-ground parts of plants, is one of the largest habitats on Earth (1). Phyllosphere microbes are diverse and serve important functions for plants (2). For instance, bacteria in the phyllosphere can have inter-kingdom interactions with insects (3 – 6). Experiments with synthetic bacterial communities can illuminate complex ecological interactions in the phyllosphere (7, 8). We isolated diverse bacteria from pea (*Pisum sativum*) leaf surfaces to use this experimental approach. The isolates described here will enable synthetic community studies on interactions of phyllosphere microbes and insects.

In the summer of 2019, leaves were sampled from *P. sativum* plants from four gardens in Ithaca, NY, USA (N 42.4438°; W 76.4671°), washed, and sonicated with 10 mM MgCl_2_ buffer. Samples were plated individually on King’s B (KB) media and incubated at 28°C for 48 hours at ambient humidity and air conditions. The resulting single colonies were grown overnight at 28°C in liquid KB media and pelleted for DNA extraction. The Qiagen DNeasy Blood and Tissue kit was used with alterations: 200 µL PBS and 20 µL proteinase K, followed by 200 µL buffer AL, were used in cell lysis, as was bead beating with silicon beads (0.4 mm). The near-full 16S rRNA gene was then PCR amplified using primers 27F (AGAGTTTGATCMTGGCTCAG) and 1492R (GGTTACCTTGTTACGACTT), and then the forward read only was sequenced (~1,000 bp). Based on BLAST results with this 16S sequence as a query, we identified isolates to the genus level and chose eight isolates from eight separate plants across the four locations to represent the diversity of bacteria commonly found in phyllosphere communities (9).

Libraries were prepared as described by Baym et al. (10), and Illumina sequencing with the NextSeq 550 platform took place at the Microbial Genome Sequencing Center (MiGS: Pittsburgh, now called the SeqCenter) with paired-end reads (2 × 151 bp) (Table 1). Raw reads were filtered for quality and adaptors using FastQC (v0.11.8) (11), then assembled with isolateSPAdes (v3.15.2) (12). Small contigs (<200 bp) and contaminant contigs were removed from assemblies.

**TABLE 1:** Summary of genome sequencing results

Strain	Taxon	Gram-	Genome size (bp)	GC%	Final coverage	No. of raw reads	No. of contigs	Contig *N*_50_ value	No. CDS	Completeness^a^	GenBank accession^b^	Short Read Archive accession
PsM3	*Arthrobacter* sp.	Positive	4,692,878	66.5	75	3,017,448	197	94,477	4,287	99.71	JARUXE000000000	SRR24496259
PsM8	*Curtobacterium* sp.	Positive	3,633,411	71	57	1,680,143	248	40,607	3,416	98.93	JARUXD000000000	SRR24496258
PsM10	*Agreia* sp.	Positive	4,010,593	67	106	3,720,303	107	292,869	3,761	99.24	JARUXC000000000	SRR24496257
PsM16	*Bacillus* sp.	Positive	3,729,660	41	113	3,572,660	190	516,346	3,754	99.59	JARUXB000000000	SRR24496256
PsM26	*Sphingomonas* sp.	Negative	5,387,916	61.5	65	3,435,182	512	157,928	4,983	99.30	JARUXA000000000	SRR24496255
PsM31	*Erwinia* sp.	Negative	4,841,466	55.5	65	3,749,646	175	150,708	4,424	99.99	JARUWZ000000000	SRR24496254
PsM32	*Paenibacillus* sp.	Positive	5,401,534	38.5	75	4,381,432	136	321,196	4,696	99.45	JARUWY000000000	SRR24496253
Pan8	*Pantoea agglomerans*	Negative	4,729,284	55	86	3,732,171	56	217,580	4,303	100	JARUWX000000000	SRR24496252

^a^ Completeness as a percentage score obtained from CheckM v.1.2.2 using the lineage workflow with default parameters (13).

^b^ GenBank accessions annotated by the NCBI PGAP pipeline (v6.5) (14).

**Fig 1:**
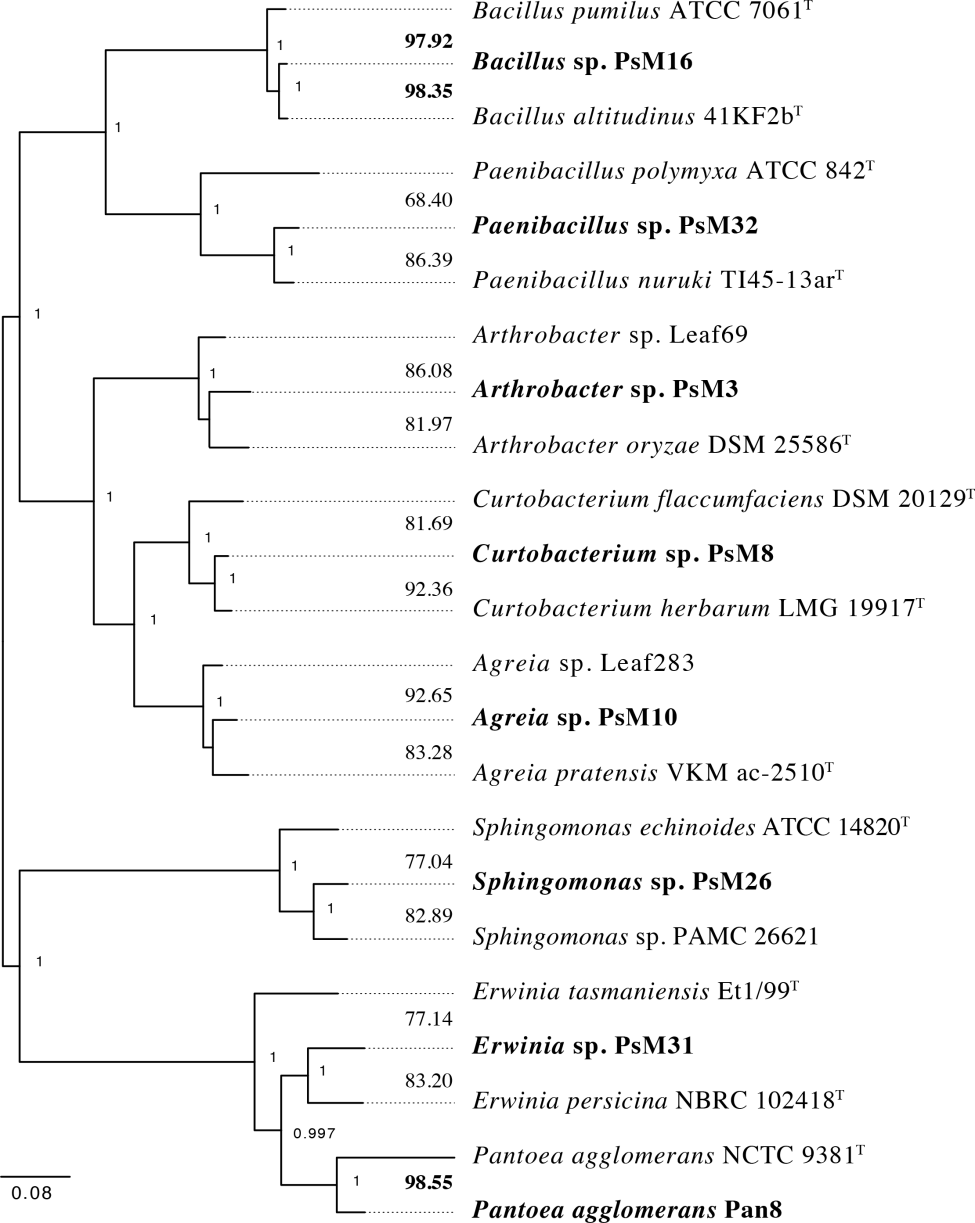
Maximum likelihood phylogenomic tree of newly sequenced phyllosphere isolates constructed with PhyloPhlAn (v3.0). The default PhyloPhlAn database for amino acid sequences was used to identify conserved protein sequences, sequence alignment used MAFFT (v7.520) (18), and phylogenetic reconstruction used RAxML (v8.2.12) (19) with program chosen models (fixed base frequency and LG substitution matrix) and 10,000 bootstrap replicates. Isolates from this study are in bold, ANI values between each isolate and the most similar previously sequenced genomes are given at the tips. ANI values in bold show species level (>95%) similarity with known species. Bootstrap values above 0.7 are shown at nodes. Closely related genomes were acquired from GenBank: *Bacillus pumilus* (GenBank accession no. ABRX00000000), *Bacillus altitudinus* (ASJC00000000), *Paenibacillus polymyxa* (CAIGJZ00000000), *Paenibacillus nuruki* (MDER00000000), *Arthrobacter* sp. (LMLR00000000), *Arthrobacter oryzae* (RBIR00000000), *Curtobacterium flaccumfaciens* (CP080395), *Curtobacterium herbarum* (JANVAG00000000), *Agreia* sp. (LMNF00000000), *Agreia pratensis* (FXAY00000000), *Sphingomonas echinoides* (AHIR00000000), *Sphingomonas* sp. (AIDW00000000), *Erwinia tasmaniensis* (CU468135), *Erwinia persicina* (BCTN00000000), and *Pantoea agglomerans* (UGSO00000000 UGSO00000000).

For analysis, genomes were annotated by RAST (RASTtk, processed in July 2021) (15). For each genome, the full length 16S rRNA gene was extracted from RAST annotations and used to query the GenBank nonredundant nucleotide collection, RefSeq representative genomes, and whole-genome shotgun contig databases. Representative genomes for all species with ≥98% 16S identity were included in pairwise average nucleotide identity (ANI) comparisons using BLAST in the JspeciesWS online calculator (v4.0.2) (16). The two genomes with the highest ANI values compared to each isolate were used in phylogenomic reconstruction using PhyloPhlAn (v3.0) (17) (Fig. 1). Unless otherwise stated, default parameters were used for all software.

Based on ANI comparisons, six of the eight isolates are undescribed species (Fig. 1). These genomes are relatively complete (98.93%–100%) and range from 3.63 to 5.40 Mb in size (Table 1). These genera are frequently found in the phyllosphere (9). These isolates provide a phylogenetic breadth for use in experimental studies.

## Data Availability

Raw sequencing reads are available from the NCBI Short Read Archive (SRA) under the BioProject accession PRJNA937792.
